# Evaluation of Brain and Cervical MRI Abnormality Rates in Patients With Systemic Lupus Erythematosus With or Without Neurological Manifestations

**DOI:** 10.5812/kmp.iranjradiol.17351065.3138

**Published:** 2011-11-25

**Authors:** Mohammad Hossein Harirchian, Hazhir Saberi, Seyed Reza Najafizadeh, Seyed Ali Hashemi

**Affiliations:** 1 Department of Neurology, Imam Khomeini Hospital, Iranian Center of Neurological Research, Tehran University of Medical Sciences, Tehran, Iran; 2 Department of Radiology, Advanced Diagnostic and Interventional Radiology Research Center (ADIR), Imam Khomeini Hospital, Tehran University of Medical Sciences, Tehran, Iran; 3 Department of Rheumatology, Imam Khomeini Hospital, Tehran University of Medical Sciences, Tehran, Iran

**Keywords:** Lupus Erythematosus Systemic, Brain, Magnetic Resonance Imaging, Neurologic Manifestations

## Abstract

**Background:**

Central nervous system (CNS) involvement has been observed in 14-80% of patients with systemic lupus erythematosus (SLE). Magnetic resonance imaging (MRI) is an appropriate method for evaluating CNS involvement in these patients. Clinical manifestations and MRI findings of CNS lupus should be differentiated from other mimicking diseases such as multiple sclerosis (MS).

**Objectives:**

The aim of this study was to evaluate the prevalence and extent of brain and cervical cord MRI lesions of lupus patients. The relationship between neurological signs and symptoms and MRI findings were evaluated as well.

**Patients and Methods:**

Fifty SLE patients who had been referred to the rheumatology clinic of our hospital within 2009 were included in a cross sectional study. All patients fulfilled the revised 1981 American College of Rheumatology (ACR) criteria for SLE. We evaluated the neurological signs and symptoms and brain and cervical MRI findings in these patients.

**Results:**

Forty-one patients (82%) were female and nine (18%) were male. The mean age was 30.1 ± 9.3 years. Twenty eight (56%) patients had an abnormal brain MRI. No one showed any abnormality in the cervical MRI. The lesions in 20 patients were similar to demyelinative plaques. Seventeen patients with abnormal brain MRI were neurologically asymptomatic. There was only a significant relationship between neurological motor manifestations and brain MRI abnormal findings.

**Conclusions:**

Unlike the brain, cervical MRI abnormality and especially asymptomatic cord involvement in MRI is quite rare in SLE patients. This finding may be helpful to differentiate SLE from other CNS disorders such as MS.

## 1.Background

Systemic lupus erythematosus (SLE) is a connective tissue disease with multi-organ involvement. In the course of the disease, inflammatory process and tissue destruction in various body organs such as the kidney, hematopoietic systems, joints, skin and central as well as peripheral nervous system are produced by autoantibodies against host antigens [1]. The clinical course is miscellaneous and unpredictable and the disease spectrum may differ from mild and prolonged manifestations to an acute life threatening illness [2][3][4].

Many SLE patients develop different neurologic symptoms within the course of their disease. Only few studies have been performed on the neurologic or neuropsychiatric involvement in SLE (NSLE, NPSLE) [5][6][7][8]. Central nervous system (CNS) involvement has been observed in 14-80% of lupus patients. The neurological manifestations of SLE are highly diverse. It could be due to vasculopathy, coagulopathy and vasculitis [6]. The pathogenesis of CNS involvement has been introduced as multiple microinfarctions, non-inflammatory thickness of small vessels with intima proliferation, small vessel obstruction and intracerebral embolism or hemorrhage. Ischemic as well as multiple sclerosis (MS) like demyelination are seen in the pathology [9]. Although it has been considered that nervous system involvement is mainly due to vasculitis, recent investigations revealed that real vasculitis is rare in lupus and the majority of patients have vasculopathy, which is manifested as mild to moderate perivascular accumulation of mononuclear cells without destruction (fibrinoid necrosis) of the blood vessels. Vessel hyalinization, perivascular lymphocytosis and proliferation have been detected in vasculopathy [9].

Magnetic resonance imaging (MRI) is an appropriate method for evaluating CNS involvement in these patients. Clinical manifestations and MRI findings of CNS lupus could be mistaken with other mimicking diseases such as MS. It has been shown that asymptomatic cervical involvement in MRI of SLE patients is quite rare [5].

## 2. Objectives

The objective of our study was to evaluate and determine the frequency of CNS (brain and cervical) clinical as well as imaging (MRI) involvement in our SLE patients.

## 3. Patients and Methods

A total of 50 lupus patients who were referred to the rheumatology clinic of our center within 2009 were evaluated in a cross sectional study. All patients fulfilled the revised 1981 American College of Rheumatology (ACR) criteria for SLE [10][11]. The sample size was calculated equal to 50 because the prevalence of detected abnormalities in the MRI of SLE patients has been reported as 70% according to Cotlon’s study [5] with a type 1 error of 0.05 and accuracy of 0.12.

All of the patients with SLE, neurologically symptomatic or asymptomatic, completely underwent neurological examination. Patients in whom other concomitant disorders caused the neurological manifestations were excluded. Brain and cervical MRI were performed for all lupus patients with a GE 1.5 Tesla machine. The thickness and distance of images were 4 and 0.5 millimeters, respectively. We used Magnevist to evaluate the enhancement. We evaluated the neurological signs and symptoms and brain and cervical MRI findings in these patients. The mean and standard deviation have been used for quantitative variables and frequency and rate have been used for qualitative variables. Chi square test and t test were used for comparison of frequencies and means as well. A P < 0.05 was considered statistically significant. SPSS statistical software version 11.5 was used for data analysis.

## 4. Results

Forty-one patients (82%) were female and nine (18%) were male with the age (mean ± SD) of 30.1 ± 9.3 and range of 16-55 years. The mean of SLE duration was 5.9 ± 4.9 years with the range of 1 month to 25 years. Fifteen (30%) of the patients had at least one neurologic sign and symptom through their disease course. Regarding MRI findings, 28 (56%) patients had an abnormal brain MRI. None of the patients had cervical lesions. The location of the lesions are shown in Table 1.

**Table 1:** MRI Findings Based on the Location of the Lesions

	**Frequency**	**%**
Subcortical	17	34
Periventricular	10	20
Cerebellum	3	6
Brain stem	2	4
Occipital cortex	1	2

According to the type of brain MRI abnormality, 20 (40%) patients had hyperintense focus in T2 weighted and FLAIR which was similar to MS lesions, 17 (34%) had brain atrophy, one (2%) had hypointense focus in T1 and hyperintense focus in T2 and FLAIR in the occipital cortex and white matter. Most of the lesions were undetermined bright objects (UBOs) with round, oval and unformed shapes in 70, 11 and 19 percent of them. The mean of brain lesion numbers was 5 ± 2.7 with a median of five lesions. The mean of brain lesion size was 4.85 ± 2.5 mm with a median of 5 mm. None of the patients with abnormal MRI did fulfill Barkhof criteria for MS. Clinical findings based on brain MRI findings have been shown in Table 2. Among the 15 patients who were neurologically symptomatic, 11 (73.4%) had an abnormal MRI. Of the total 35 patients without neurological manifestations, MRI was abnormal in 17 (48.6%). Comparison of these rates showed no significant difference (P = 0.107) (Table 3). Among 42 female patients, MRI was abnormal in 24 (57.1%) whereas among eight male patients, four (50%) patients had an abnormal MRI. There was no significant difference between these frequencies (P = 0.71).

**Table 2:** Clinical Findings Based on Brain MRI Findings

	**Normal Brain MRI a, ** **No. (%)**	**Abnormal Brain MRI, No. (%)**	**P value b**
Cranial nerves involvement			0.128
Negative	21 (44.7)	26 (55.3)	
Positive	0	3 (100)	
Motor involvement			0.030
Negative	26 (56.6)	20 (43.4)	
Positive	0	4 (100)	
Sensory involvement			0.118
Negative	27 (56.2)	21 (43.8)	
Positive	0	2 (100)	

^a^ Abbreviation: MRI, Magnetic resonance imaging

^b^ Fisher exact test

**Table 3:** MRI Findings According to Lesion Size

	**Frequency**	**Frequency in Total Patients, %**	**Frequency in the Patients With Abnormal MRI a, %**
1-5 mm	18	36	85.7
6-10 mm	10	20	47.6
≥ 11 mm	3	6	14.2

^a^ Abbreviation: MRI, Magnetic resonance imaging

MRI findings according to lesion size have been summarized in Table 3. Mean of disease duration was 5.9 ± 5.8 and 6 ± 4.2 years in the patients with abnormal and normal brain MRI, respectively which showed no statistically significant difference (P = 0.314, t test).Thumboo J, Sunku J,

## 5. Discussion

Nowadays, along with advances in the novel radiological techniques and applying MRI, asymptomatic nervous system involvement in SLE patients may be found. MRI is the technique of choice for evaluating patients with neuropsychiatric manifestations.

In our study, NSLE was seen in 30% of the patients. In some previous studies, the prevalence of NPSLE has been reported as 14-80% with an extended spectrum [12][13]. Four of the patients had been newly diagnosed as SLE and two of them had neurological manifestations. Indeed, 4% of our patients had presented with neurological manifestations. In other studies this frequency was reported as 28-40%. This finding shows that cerebral involvement in lupus may occur during or before diagnosis [14]. The most common neurological symptoms in our patients were headache (14%), limb weakness (8%) and seizure (6%). The most common neurological signs were hemiparesis (8%), cranial nerve involvement (6%) and sensory involvement (4%). The frequency of these signs and symptoms were lower than previous studies [6].

No significant association was found between MRI abnormalities and disease duration in our study. This finding indicates that brain involvement in lupus may occur any time in the disease course. Therefore, all patients with SLE, with or without neurological manifestations are at risk of brain lesions which could be detected in MRI. In addition, we could not find a significant association between gender and abnormal MRI findings. The reason of this subject was the small sample size. Further studies in this field with larger sample sizes are helpful to achieve more definite results. The results of our study showed a significant association between hemiparesis and positive MRI findings. There was a near to significant level of association between cranial nerves and sensory disorders with MRI abnormality. Cervical cord MRI was normal in all the patients. None of the patients had cervical cord atrophy. This finding indicates that cord involvement is uncommon in this disease. It could be concluded that unlike the brain, asymptomatic cord involvement is quite rare in SLE. This point may help in differentiating SLE from other diseases such as MS. On the other hand, if a patient has MRI findings and clinical manifestations suggestive of MS, lupus should be considered, especially if cervical MRI is negative. If brain MRI in a patient raises the suspicion for MS or lupus, an asymptomatic lesion in cervical MRI is more suggestive for MS rather than lupus. It should also be kept in mind that none of the patients with abnormal MRI did fulfill Barkhof criteria and this finding could be helpful in differentiating SLE from MS.
